# Mycoflora and Natural Incidence of Selected Mycotoxins in Rabbit and Chinchilla Feeds

**DOI:** 10.1100/2012/956056

**Published:** 2012-05-03

**Authors:** Mariana Vanesa Greco, Alejandro Guillermo Pardo, Vanesa Ludemann, Pablo Eduardo Martino, Graciela Noemí Pose

**Affiliations:** ^1^Departamento de Ciencia y Tecnología, Universidad Nacional de Quilmes (UNQ), Roque Sáenz Peña 352, B1876BXD Bernal, Provincia de Buenos Aires, Argentina; ^2^Consejo Nacional de Investigaciones Científicas y Técnicas (CONICET), Avenida Rivadavia 1917, C1033AAJ CABA, Argentina; ^3^Facultad de Ciencias Veterinarias, Universidad Nacional de la Plata, Calle 60 y 118 S/N, 1900 La plata, Buenos Aires, Argentina; ^4^Comisión de Investigaciones Científicas de la Provincia de Buenos Aires (CIC), Calle 526 entre 10 y 11, 1900 La Plata, Buenos Aires, Argentina; ^5^Universidad Nacional de Río Negro, Mitre 331, 8336 Villa Regina, Río Negro, Argentina

## Abstract

Mycotoxins are secondary metabolites produced by filamentous fungi that cause a toxic response when ingested by animals or man. Demand of natural fur, such as those from rabbit and chinchilla, produced under controlled conditions, has increased worldwide. The toxicogenic mycoflora contaminating feeds for these animals was enumerated and identified. Six of the major mycotoxins implicated in animal mycotoxicosis were detected and quantified. Moulds count ranged from <10 to 4.7 × 10^5^ CFU g^−1^; 14% of the samples exceeded the limit that determines hygienic feed quality. More than twenty species belonging to the five most important mycotoxigenic mould genera were recovered. Among the analyzed mycotoxins, aflatoxins were recovered in 100% of the examined samples, deoxynivalenol in 95%, fumonisins in 100%, ochratoxin A in 98%, T2 toxin in 98%, and zearalenone in 100%. Cooccurrence of mycotoxins was observed in 100% of the samples analyzed. Exposure to multiple mycotoxins was thus demonstrated for these animals.

## 1. Introduction

There is an increasing worldwide demand of natural fur produced under controlled conditions. Among them, chinchilla, fox, mink, and otter fur are quite appreciated. On the other hand, rabbit breeding besides fur provides meat intended for human consumption [1]. Worldwide production of rabbit meat was 1.1 million tons per year between 2002 and 2005 and has grown around 49% over the last 15 years with China, Italy, Spain, and France being the main producers [2]. In America, Argentina is the main producer and exporter. In 2004 Argentina exported 1400 tons of rabbit meat to the EU [2]. 

 Commercial feedstuffs are the main consumables in rabbit and chinchilla breeding representing 60–70% of production cost [3]. Filamentous fungi are ubiquitous in nature and responsible for producing mycotoxins in agricultural crops [4]. Rabbit and chinchilla feed ingredients that constitute complete feed products are derived from different raw materials. Inadequate management of raw materials can lead to undesirable growth of fungi, leading to loss of nutritive substances and resulting in contamination by mycotoxins [5]. Lowered production, illness, and death can be consequences of mycotoxin-contaminated feeds [6].

 One of the main features of mycotoxicosis in rabbits is feeding reduction (about 20–60%) which causes a delay in growth and thus reduced productivity. Mycotoxicosis in rabbits includes acute or chronic pathologies depending on the mycotoxin involved, its concentration, period of exposure, accumulative effects, and synergisms among mycotoxins. In some cases abortion and death of adults may occur [7]. Also, it is necessary to consider that even though there is little information available regarding the prevalence and concentration of mycotoxins in foods of animal origin, some toxins ingested by animals may be found in meat, milk, or eggs [8]. Regular monitoring of toxigenic mycoflora of the agriculturally based feeds and foods is an essential pre-requisite for development of strategies to control or prevent mycotoxin exposure of animal and human populations [9]. 

 Despite the great attention that has been paid to the study of toxicogenic moulds and their mycotoxins in various feeds, little is known about fungal and mycotoxin contamination of mixed chinchilla and rabbit feed. Thus, the aim of this work was to study the toxicogenic mycoflora including enumeration and identification of mould genera and species naturally contaminating feeds for these animals along with detection and quantification of the major mycotoxins: aflatoxins, ochratoxin A, T-2 toxin, fumonisins, deoxynivalenol (DON), and zearalenone.

## 2. Materials and Methods

### 2.1. Sample Collection and Preparation

 A total of 42 representative samples (1-2 kg per sample) of finished rabbit (17) and chinchilla (25) feeds were collected from 7 companies in 5 provinces of Argentina (Buenos Aires, Córdoba, La Pampa, La Rioja, and Mendoza) in 2010. All samples were homogenized and divided to obtain a 1 kg working sample for analysis. Each sample was ground in a laboratory mill. For mycological examination feed samples were immediately analyzed upon arrival or they were stored for 2-3 days in paper bags at room temperature (about 25°C). Feed samples intended for mycotoxin analysis were stored at −20°C.

### 2.2. Mycological Analysis

 The dilute plate technique was used for enumeration and isolation of fungi [11]. Ten grams of each milled feed sample was mixed with 90 mL of 0.1% peptone and shaken on a horizontal shaker for 20 minutes. Then, 0.1 mL of a proper spore suspension dilution (made up to 10^5^ spores per mL) was inoculated onto the following media: dichloran rose bengal chloramphenicol agar (DRBC) to enumerate total culturable fungi, dichloran 18% glycerol agar (DG18) to enumerate xerophilic fungi, and dichloran chloramphenicol peptone agar (DCPA) for selective isolation of *Alternaria* and *Fusarium* species [11]. Plates were incubated at 25°C for 7 days. The DCPA plates were incubated under a 12 h of light: 12 h of darkness photoperiod. For counting, plates containing 10–100 colonies were used and the results were expressed as colony-forming units per gram of sample (CFU g^−1^) [11]. Individual CFU g^−1^ counts for each colony type, considered to be different, were recorded. Representative colonies of each type were transferred for sub-culturing onto plates with malt extract agar (MEA) or water agar (WT), for moulds suspected to belong to *Alternaria* or *Fusarium* genera. Filamentous fungi were identified at genus level according to macro- and microscopic criteria in accordance with Samson et al. [12]. Fungal isolates were identified at species level according to the leading authorities: *Penicillium *and* Aspergillus spp.* according to Pitt and Hocking [11], *Fusarium spp.* according to Nelson et al. [13], *Alternaria spp.* according to Simmons [14], and other fungi according to Pitt and Hocking [11]. The isolation frequency (Fr) and relative density (RD) of genus/species were calculated according to González et al. [15], Pacin et al. [16], and Saleemi et al. [9] as follows:

Fr (%) = number of samples with a genus or species/total number of samples × 100.

RD (%) = number of isolates of a genus or species/total number of fungi isolated × 100.

All the isolates were preserved on agar slants of malt extract agar (MEA) or potato dextrose agar (PDA) for *Alternaria* and *Fusarium* at 4°C and cryopreserved in 18% glycerol at −20°C. 

### 2.3. Mycotoxin Analysis

 To evaluate mycotoxin occurrence, feed samples were subjected to quantitative analyses using ELISA-based analytical test kits for aflatoxins, ochratoxin A, T-2 toxin, fumonisins, deoxynivalenol (DON), and zearalenone (RIDASCREEN FAST, R-Biopharm AG). The extraction procedures were according to manufacturer protocols. In brief, 5 g of ground sample was extracted with 25 mL of 70% methanol for aflatoxins, T-2 toxin, zearalenone, and fumonisins. For ochratoxin A and DON, samples were extracted with 12.5 mL of 70% methanol or 100 mL of distilled water, respectively. Afterwards, samples were shaken vigorously for 3 minutes and the extracts filtered through the Whatman N°1 paper. Then, aflatoxin, ochratoxin A, T-2 toxin, and zearalenone filtrates were diluted with distilled water in the ratio 1 : 1 and fumonisin filtrates in the ratio 1 : 14. Fifty *μ*L of the diluted filtrate per well were used for testing. 

## 3. Results 

 This study shows that fungi and mycotoxins were present in all the feed samples assayed. Fungal counts (CFU g^−1^) on each medium are shown in Table 1. Total fungal counts on DRBC ranged from <10 to 4.7 × 10^5^ CFU g^−1^. High fungal contamination was found in 6 out of 42 samples, exceeding the limit of 1 × 10^4^ CFU g^−1^, that determines feed hygienic quality [17]. Xerophile moulds counts ranged from <10 to 8.5 × 10^5^ CFU g^−1^. Spoilage of feedstuff can be due to xerophilic fungi, which are capable of rapid growth above about 0.77 a_*w*_ and of slow growth at 0.75 a_*w*_ and below, down to about 0.68 a_*w*_ [11]. On DCPA fungal counts ranged from <10 to 2.7 × 10^5^ CFU g^−1^.

 Both field and storage fungi were found in this study. Mycotoxigenic genera such as *Aspergillus, Penicillium, Fusarium*, and *Alternaria* were recorded. These moulds are of great importance due to potential mycotoxin production, which can contaminate many agricultural commodities like wheat, oat, barley, sunflower, soybean, and so forth, used in the formulation of finished feeds. *Cladosporium*, *Trichoderma*, and other mitosporic Ascomycetes were also found. One genus belonging to mycotoxigenic Ascomycetes, *Eurotium*, and one genus belonging to Zygomycetes, *Mucor*, were determined. The most frequent fungi were those from the genus *Eurotium*, recovered from 29 samples (Fr 69%). In the second place were moulds from the genus *Aspergillus*, recovered from 22 samples (Fr 52.4%). moulds from the genus *Cladosporium* (Fr 42.5%), *Penicillium* (Fr 33.3%), and *Mucor* (Fr 33.3%) and yeast (Fr 40.5%) were recovered with relative high frequency. In less proportion, other genera recovered were *Fusarium, Alternaria, Trichoderma, Scopulariopsis*, and *Paecilomyces* (Table 2). 

 The fungal species isolated on different agar media are shown in Table 3. This table also illustrates the frequency and relative density of recovered species. Among *Eurotium *species, *E. amstelodami* was the most prevalent (Fr 50%). Other *Eurotium spp.* recovered were *E. chevalieri, E. repens*, and *E. rubrum*. Mycotoxigenic species such as *A. flavus, A. parasiticus, P. expansum, P. roqueforti, F. proliferatum*, and* F. subglutinans* were also found in our work. 

 There is only limited data on the occurrence of important mycotoxins in rabbit feed; thus, research and surveys in this area are very important [10]. In our study we have determined that all samples were contaminated with mycotoxins (Table 4). The concentration of different mycotoxins in finished 

mixed chinchilla and rabbit feed samples is shown in Table 5. Recommended maximum amount of mycotoxins in rabbit feed is shown in Table 6 [10]. Though only one sample had aflatoxin and fumonisin concentrations exceeding the recommended limits, several toxins have been detected in low concentrations in all the samples, which might lead to a 

response of synergic toxicity in animals under this type of exposure (Table 5). 

## 4. Discussion 

 Fungal growth on raw materials used as ingredients (in the field or during silage storage) leads to contamination of the final feed. This fungal growth reduces nutritional value and may result in the production of mycotoxins, which constitute a risk factor for animal health [5, 18]. Mycotoxigenic fungi, such as those we found in our work, are undesirable because of their potential for mycotoxin production. Other fungi isolated such as *Mucor* and *Cladosporium* species may cause mycotic abortion and allergy in animals and humans [19]. 

 The genus *Eurotium* is an important mycotoxin producer. *Eurotium* species can produce echinulin, neoechinulin A, flavoglaucin, physcion, auroglaucin, dihydroauroglaucin, and tetrahydroauroglaucin [20]. Echinulin has been detected in feeds containing a high propagule density of *E. chevalieri* and *E. amstelodami*. These species were capable of producing echinulin on rice [21]. Rabbits injected intraperitoneally with purified echinulin have shown a significant degree of lung and liver damage [22]. Also, the production of aflatoxins has been reported for *E. amstelodami, E. repens*, and* E. rubrum* [23–26], and the production of ochratoxin A has been reported for *E. amstelodami* [23]. However, we should bear in mind that rabbits are one of the most sensitive animals to toxins such as aflatoxins, zearalenone, fumonisins, DON, and T-2 toxin. Aflatoxicosis in rabbits has been reported with 33–10400 ppb of aflatoxin B1 in feed. The rabb

its affected showed loss of coordination, loss of weight, and jaundice before death. Also, zearalenone affects viability of embryos and fertility. The consumption of feed contaminated with 200 ppb zearalenone produced abortion and yellow diarrhea in suckling rabbits. Furthermore, fumonisin B1 can cause multiorgan failure (i.e., kidneys, live

r, lungs, heart, brain), leukoencephalomalacia, and reduction in the fetus weight [27]. Also, T-2 toxin is hepato- and nephrotoxic in rabbits affecting reproduction as well as the digestive and respiratory systems [27]. Producers have usually been concerned with death due to diarrhea in rabbits. The DON levels of commercial feeds, particularly those containing more than 1000 ppb DON, have been blamed by some rabbit producers for this problem [10]. In our work we have shown that 13 out of 42 samples (31%) had levels of DON between 1080 and 1720 ppb. 

 Although the scientific literature offers a broad variety of information about the effects of individual mycotoxins on various animal species, concurrent exposure to multiple mycotoxins is more likely in the livestock industry. Poor livestock performance and/or disease symptoms may be due to the synergistic interactions between multiple mycotoxins [28]. In our work, cooccurrence of mycotoxins was demonstrated in 100% of the samples assayed (Tables 4 and 5). In Argentina, there is rather limited information concerning natural occurrence of mycotoxins in feedstuff, particularly with respect to rabbit feed. Only one work reported that 25% of rabbit feed samples from Córdoba province were contaminated with ochratoxin A with a mean level of 21.8 ppb [29]. Also there is scarce information from other parts of the world. Mohanamba et al. [30] reported that 77% of rabbit feed samp

les were contaminated with aflatoxins in India. 

## 5. Conclusions 

 The present study has provided information about the contaminating toxigenic mycoflora in rabbit and chinchilla feeds in Argentina. This is the first report describing the cooccurrence of six mycotoxins. These toxic substances are known to be either carcinogenic, neurotoxic, nephrotoxic, dermatotoxic, or immunosuppressive. Although the synergic effects of mycotoxins on health and productivity of other animal species such as poultry have been well documented [31], more studies are needed in order to screen the presence of different mycotoxins in different feeds. Particular attention should be paid to the cooccurrence and synergic effects of mycotoxins present in low levels in order to avoid the consumption of contaminated feeds which could provoke acute or chronic illnesses leading to economic losses. 

## Figures and Tables

**Table 1:** Fungal counts (CFU g^−1^) from chinchilla and rabbit feed samples.

Parameters	DRBC	DG18	DCPA
No. of samples tested	42	42	42
Less count (CFU g^−1^)	<10	<10	<10
Highest count (CFU g^−1^)	4.7 × 10^5^	8.5 × 10^5^	2.7 × 10^5^
Average count (CFU g^−1^)	3.34 × 10^4^	7.34 × 10^4^	3.02 × 10^4^
Median count (CFU g^−1^)	5 × 10^2^	1.35 × 10^3^	8 × 10^2^
No. of samples exceeding the limit of hygienic quality	6 (17%)	12 (33%)	7 (19%)

**Table 2:** Fungal genus present in chinchilla and rabbit feed samples.

Genus	Number of isolates	Fr (%)*	Rd (%)**
*Eurotium *	30	71.43	20
*Aspergillus *	21	50	14
*Cladosporium *	21	50	14
*Penicillium *	14	33.3	9.5
*Mucor *	14	33.3	9.5
*Paecilomyces *	4	9.5	2.7
*Fusarium *	3	7.1	2.1
*Trichoderma *	3	7.1	2.1
*Scopulariopsis *	3	7.1	2.1
*Alternaria *	1	2.4	0.7
Others	16	38	11
Yeast	18	42.9	12

*Isolation frequency.

**Isolation relative density.

**Table 3:** Fungal species present in chinchilla and rabbit feed samples.

Species	Number of isolates	Fr (%)*	Rd (%)**
*Eurotium amstelodami*	21	50.00	15.44
*E. chevalieri*	11	26.19	8.09
*E. repens*	13	30.95	9.56
*E. rubrum*	8	19.05	5.88
*Eurotium sp.*	7	16.67	5.15
*Aspergillus candidus*	2	4.76	1.47
*A. flavipes*	2	4.76	1.47
*A. flavus*	3	7.14	2.21
*A. niger*	1	2.38	0.74
*A. parasiticus*	2	4.76	1.47
*A. penicillioides*	2	4.76	1.47
*A. terreus*	1	2.38	0.74
*A. versicolor*	2	4.76	1.47
*Aspergillus sp.*	9	21.43	6.62
*Cladosporium cladosporioides*	21	50.00	15.44
*Penicillium brevicompactum*	2	4.76	1.47
*P. expansum*	8	19.05	5.88
*P. funiculosum*	1	2.38	0.74
*P. olsonii*	1	2.38	0.74
*P. roqueforti*	1	2.38	0.74
*Penicillium sp.*	2	4.76	1.47
*Fusarium proliferatum*	3	7.14	2.21
*F. subglutinans*	1	2.38	0.74
*Alternaria tenuissima*	1	2.38	0.74
*Paecilomyces variotii*	4	9.52	2.94
*Scopulariopsis brevicaulis*	3	7.14	2.21
*Trichoderma harzianum*	3	7.14	2.21
*Mucor sp.*	1	2.38	0.74

*Isolation frequency.

**Isolation relative density.

**Table 4:** Number of samples tested, number of positive samples, and percentage and levels of detected mycotoxins.

Parameter	Mycotoxins (ppb)					
	Aflatoxins	Deoxynivalenol	Fumonisins	Ochratoxin A	T2 toxin	Zearalenone
No. of samples tested	42	42	42	42	42	42
No. of positive samples	42	40	42	41	41	42
Percentage positive (%)	100%	95%	100%	98%	98%	100%
Range (ppb)	<1.70–22.55	222–1740	222–6000	<5–26.57	<50–129.88	<50–177.97
Median (ppb)	7.26	743	462	9.74	50	50
Highest level (ppb)	22.55	1740	6000	26.57	129.88	177.97

**Table 5:** Concentration of des. ND: not detected; CH: chinchilla; R: rabbit.

Mycotoxins (ppb)						
Samples tested	Aflatoxins	Deoxynivalenol	Fumonisins	Ochratoxin A	T2 toxin	Zearalenone
CH1	3.91	393	222	5	50	50
CH2	1.84	ND	222	8.09	50	50
CH3	1.7	ND	222	9.74	ND	50
CH4	6	737	222	7.77	50	50
CH5	6.08	761	331	7.83	50	50
CH6	7.22	1592	222	6.24	50	50
CH7	7.19	222	270	6.53	50	50
CH8	5.73	610	795	12.48	50	50
CH9	5.55	1080	598	10.17	50	80.17
CH10	4.26	1210	370	8.95	50	50
CH11	6.24	868	525	8.66	50	50
CH12	7.64	629	732	8.6	50	50
CH13	8.6	488	503	13.19	50	50
CH14	8.77	409	370	10.23	50	50
CH15	9.24	760	866	23.49	50	82.12
CH16	7.09	1210	222	23.49	50	68.69
CH17	6.92	1300	222	15.25	50	75.59
CH18	7.5	548	626	12.63	50	53.96
CH19	8.77	937	1030	11.81	50	50
CH20	8.24	798	715	23.34	50	50
CH21	10.17	1720	222	25.38	50	58.34
CH22	9.46	241	447	11.59	50	66.49
CH23	6.67	1390	975	16.91	50	79.9
CH24	10.98	1660	498	11.59	50	78.81
CH25	9.15	462	340	10.9	50	71.2
R1	22.55	327	6000	5	50	50
R2	3.66	222	236	ND	81.75	50
R3	1.7	355	222	5	50	50
R4	1.99	279	222	5	50	50
R5	7.2	222	3110	6.6	50	50
R6	5.57	256	1500	5.76	50	50
R7	7.46	222	376	5.55	50	50
R8	9.72	916	562	5	50	50
R9	9.93	1300	953	17.44	50	50
R10	9.97	1100	581	26.57	50	50
R11	7.96	700	709	8.43	129.88	50
R12	6.14	1190	477	8.54	120.27	50
R13	7.14	749	241	16.81	128.98	50
R14	9.6	222	786	12.18	50	177.97
R15	7.94	1740	311	13.11	50	140.38
R16	9.19	1690	390	9.54	50	105.36
R17	7.29	467	492	7.01	50	50

**Table 6:** Recommended maximum concentration of mycotoxins in rabbit feed [10].

Mycotoxin	Maximum content for feed with a moisture content of 12%	
	ppm (mg/kg)	ppb (*μ*g/kg)
Aflatoxin B1	0.02	20
Ochratoxin A	5	5000
Deoxynivalenol	5	5000
Zearalenone	0.50	500
Fumonisin B1 + B2	5	5000
